# Identification of B Cell Subpopulations with Pro- and Anti-Tumorigenic Properties in an Immunocompetent Mouse Model of Head and Neck Squamous Cell Carcinoma

**DOI:** 10.3390/cells14010020

**Published:** 2024-12-29

**Authors:** Michael Sonntag, Sandra Stanojevic, Simon Laban, Patrick J. Schuler, Thomas K. Hoffmann, Cornelia Brunner

**Affiliations:** 1 Department of Otorhinolaryngology, Ulm University Medical Center, 89075 Ulm, Germany; michael.sonntag@uniklinik-ulm.de (M.S.); sandra.stanojevic@uni-ulm.de (S.S.); simon.laban@uniklinik-ulm.de (S.L.); patrick.schuler@uniklinik-ulm.de (P.J.S.); t.hoffmann@uniklinik-ulm.de (T.K.H.); 2 Department of Otorhinolaryngology, University Hospital Heidelberg, 69120 Heidelberg, Germany; 3 Core Facility Immune Monitoring, Medical Faculty, Ulm University, 89075 Ulm, Germany

**Keywords:** B cells, HNSCC, germinal centers, adenosine, immunocompetent mouse model

## Abstract

Due to their high developmental diversity and different regulatory and functional roles, B cell subpopulations can promote or inhibit tumor growth. An orthotopic murine HNSCC model was applied to investigate the B cell composition and function in HNSCCs. Using flow cytometry approaches, cells from the spleen, lymph nodes and tumors were analyzed. Additionally, immunoglobulin (Ig) levels post-tumor induction were tracked via enzyme-linked immunosorbent assays (ELISA). Following tumor induction, GCs, as well as increasing numbers of GL7^+^CD95^+^ GC B cells in the spleen and tumor tissues, were detected. In parallel, we observed CD39^+^CD73^+^ B cells in tumors and spleens of tumor-bearing mice. Notably, CD39^+^CD73^+^ expression was primarily detected on MZ B cells and to a lesser extent on follicular (FO) and non-follicular, newly formed (NF) B cells, supposing an immunosuppressive function of MZ B cells in the TME. Parallel to increased MZ B cell numbers in secondary lymphoid organs (SLOs) as well as in the tumor tissue, IgM antibody (Ab) levels rose continuously. In contrast, IgG1, IgG2, and IgG3 levels increased at later time points. Understanding the complex interactions between B cell subsets and the TME could lead to new strategies for enhancing the treatment and prognosis of HNSCC patients.

## Simple Summary

Head and neck squamous cell carcinoma (HNSCC) is a very heterogeneous cancer entity with an unsatisfactory prognosis. Despite intensive research and the use of new therapeutic options such as immunotherapy, the 5-year survival rate is not higher than 50% for male individuals, meanly affected by HNSCC, and has not been significantly improved over the last 20 years. The immunosuppressive microenvironment, which is characteristic of head and neck tumors (HNCs), contributes significantly to the failure of new therapy concepts. Therefore, the aim of this work was to identify pro- and anti-tumorigenic immune cell populations in the HNSCC tumor environment with a special focus on the B cell subsets, as recent data assign B lymphocytes a significant contribution to tumorigenesis. In addition to an increase in anti-tumorigenic germinal center (GC) B cells, we describe here for the first time the population of marginal zone (MZ) B cells in the tumor microenvironment (TME) of HNSCC with pro-tumorigenic potential.

## 1. Introduction

### 1.1. Understanding the Complex Landscape of HNC: Heterogeneity, Impact, and Prognosis

HNCs are a heterogeneous group of aggressive malignant neoplasms [1] originating from the upper aerodigestive tract, with 90% arising from epithelial tissues in the oral and pharyngeal cavities [2]. HNC is the seventh most common cancer globally, accounting for more than 660,000 new cases and 325,000 deaths annually [3,4]. Over 95% of HNCs are HNSCCs of the oral cavity, pharynx, and larynx. The etiology and incidence of HNSCCs are undergoing significant changes, with distinct trends observed across cancer subtypes [5]. Historically driven by tobacco and alcohol, laryngeal cancer incidence has declined alongside reduced smoking rates [5]. In contrast, oropharyngeal cancer incidence has tripled over the past two decades, particularly in younger populations, largely attributed to human papillomavirus (HPV) infection [5]. Notably, a subset of oral cancers in nonsmokers and non-drinkers, particularly among women, suggests an emerging distinct etiology [5,6]. While mutagen-associated HNCs are declining, there is a rise in HPV-associated oropharyngeal and EBV-associated nasopharyngeal carcinomas [7]. HNSCCs exhibit inter- and intratumoral heterogeneity, impacting therapeutic responses [8]. Despite advanced treatments, HNSCCs have a poor prognosis, with a 5-year survival rate of 63% for females and 47% for males. At the time of diagnosis, approximately two-thirds of patients present with a locally or regionally advanced tumor stage, which is associated with a higher likelihood of distant metastases and post-therapeutic relapses [9]. Intensive research is required to understand the TME and immune system interactions to develop effective anti-tumor therapeutics.

### 1.2. Exploring the TME in Orthotopic Grown Tumors

Initial studies have shown differences in growth patterns between orthotopic and ectopic tumors in xenograft models [10]. To simulate localized tumor growth in HNCs, potentially recapitulating tissue-specific tumor-stroma-immune cell interactions, and capturing dynamic interplays between the tumor and the murine immune system concerning tumor growth and progression, SCC-VII cell line-derived murine tumor cells were injected into the oral cavity floor of C3H/HeN mice. This method resulted in consistent tumor growth across multiple trials [11,12,13,14,15], creating a physiological TME. The SCC VII cell line has been generated from a spontaneously occurring squamous cell tumor at the head and neck region from C3H mice, which was analyzed for growth rate and histological features in vitro, followed by in vivo experiments in C3H/HeJ mice in head and neck region [16]. The syngeneic C3H-SCC VII system is a widely used and accepted model for research on HPV-negative HNCs concerning the oral cavity [17,18]. This model closely mirrors the progression of human oral cancer, providing a valuable tool for research on primary and metastatic disease in HNC [16]. The optimized model using orthotopic implantation in C3H/HeN mice reflects key aspects of human HPV-negative HNSCCs and offers a robust platform for immunological studies [13]. The use of this model allows observation of tumor cell formation, stromal cells, tumor-infiltrating lymphocytes (TILs), and the mechanisms employed by tumors to evade immune surveillance within the immediate vicinity of the TME.

### 1.3. Tumorigenesis and Immune Evasion in TME

Tumor development is marked by genetic instability, with mutations and chromosomal aberrations driven by an activated, proinflammatory stroma. Tumorigenesis involves complex interactions between healthy and malignant cells, resulting in the formation of a TME surrounding the neoplastic focus. Cancer cells manipulate the host immune system to evade immune surveillance, complicating tumor-targeted therapies. Tumor cells evade immune surveillance in different ways, including generating immunosuppressive extracellular Ado within the TME [19]. Recently, adenosine (Ado)-producing regulatory B cells (Breg) have emerged as a novel immunosuppressive subset [15]. These cells, through surface ectonucleotidases CD39 and CD73, convert adenosine triphosphate (ATP) to immunosuppressive Ado, promoting tumorigenesis [12,15,20].

### 1.4. Unveiling B-Cell Complexity: Shaping Immune Responses and Impact on Tumor Dynamics in HNSCCs

Traditionally, B cells are known for modulating immune responses, producing antibodies, presenting antigens to activate T cells, and aiding T-cell proliferation [21]. B cells develop from a hematopoietic stem cell (HSC) in the bone marrow (BM) initially via pre-B and pro-B cells into immature B cells, the latter expressing a functional antigen receptor, and develop further into transitional B cells that are no longer dependent on stromal support. Transitional B cells leave the BM and migrate to secondary lymphoid organs for further maturation. There, they develop into MZ B cells and FO B cells. MZB is characterized by the production of high amounts of IgM after encounters with blood-borne, T-cell-independent antigens. Follicular B cells instead need T cell help and develop within GC after class switch recombination and affinity maturation either to memory cells or to plasmablasts that still divide and further to short-lived extrafollicular plasma cells, both producing antigen-specific antibodies (for review, see [22]), present in the tumor-microenvironment of HNSCC and contribution to anti-cancer immunity (for review see [23].

However, distinct B cell subpopulations have been discovered that contribute to immune tolerance and suppression, secreting anti-inflammatory mediators like IL-10 and Ado or expressing inhibitory molecules such as PD-L1 [15,24]. The interplay among these B cell subgroups, with pro- or anti-tumorigenic properties, influences tumor development through Ab secretion, antigen presentation, cytokine release and surface molecule expression [25]. While B-TILs in the TME are linked to favorable prognoses in various tumors, their precise role in HNSCC is uncertain due to the significant plasticity and heterogeneity of B-cell subgroups [26]. Recently, our team highlighted the pivotal role of B cells in tumor development, positioning them as viable candidates for immunotherapy [12,15]. Here, we comprehensively elucidate the immunological consequences of the generated HNSCC in the mouse model, focusing on cellular alterations specifically related to B cell subpopulations within the spleen, lymph nodes, and the tumor itself, with emphasis on B cells pro- and anti-tumorigenic properties.

## 2. Materials and Methods

### 2.1. SCC VII—Cell Culture

SCC VII cells were cultured in complete Roswell Park Memorial Institute (RPMI) 1640 medium (Gibco, Carlsbad, CA, USA) supplemented with 10% heat-inactivated fetal bovine serum (FBS, Biochrom, Berlin, Germany), 100 U/mL penicillin, and 100 mg/mL streptomycin (PAN Biotech, Aidenbach, Germany) at 37 °C with 5% CO_2_. Before injection, cultured SCC VII cells were trypsinized and washed twice with Dulbecco’s Phosphate-Buffered Saline (DPBS) medium (Gibco, Carlsbad, CA, USA).

### 2.2. Animal Model

The animal experiments in this study were approved by the regional animal ethics committee, Regierungspräsidium Tübingen, Germany (Protocol #1270). Six-week-old male C3H/HeNRj mice were obtained from Janvier Labs (Le Genest-Saint-Isle, France) and housed with four animals per cage in sterilized individual ventilated cages (IVC) under SPF conditions with ad libitum access to water and food. The housing environment was temperature-controlled with a 12-h light-dark cycle. After a two-week acclimatization period, orthotopic tumor formation was initiated by injection of 5 × 10^5^ murine SCC VII cells in Dulbecco’s Phosphate-Buffered Saline (DPBS) transorally into the floor of the mouth. Blood samples were obtained from the facial veins of mice before (day 0), as well as on days 7, 14, and 21 after tumor injection, and they were subjected to ELISA analysis. On days indicated, a defined number of tumor-bearing and control mice were euthanized, and tumor, spleen, and lymph nodes were harvested for further analysis. Tissue numbers varied due to the premature removal of animals, according to the score sheet. Tumor and spleen specimens were used for isolating TILs or splenocytes using flow cytometry or were fixed in 4% formaldehyde and embedded in paraffin for further processing.

At the start of the experiment, 4 mice were randomly selected to serve as controls. On day 0, one control mouse was harvested, and data from the spleen and blood were collected. The same procedure was performed on days 7, 14, and 21, with cervical lymph node tissue additionally collected on these days. This approach provided a single control group consisting of 4 control mice (n = 4), which served as the source for further flow cytometry (FACS) analyses using specific staining panels. The time-course data generated offered an overview of how control mice responded during the experimental period. ELISA data were derived from blood samples collected from two additional independent experiments conducted under identical conditions. Blood was drawn on all experimental days (d0, d7, d14, d21), with measurements performed for each individual mouse.

### 2.3. Spleen, Lymph Nodes, and Tumor Preparation

Abdominal and cervical lymph nodes, along with spleen tissue, were obtained and stored in 2.5 mL of RPMI medium containing 1% 100 mM sodium pyruvate, 0.1% 50 mM 2-mercaptoethanol, and 100 U/mL penicillin-streptomycin. Single-cell suspensions were prepared by passing tissues through a 40 µm EASY strainer (Greiner Bio-One, Frickenhausen, Germany). After centrifugation at 1200 rpm (=274 rcf) for 5 min, the cell pellet was resuspended in 2 mL of red cell lysis buffer (Red Blood Cell Lysis Solution (10×), Catalogue no. 130-094-183, Miltenyi Biotec, Bergisch Gladbach, Germany) and incubated at room temperature (RT) for 10 min. After the second centrifugation and removal of the supernatant, the pellet was resuspended in 5 mL of fluorescence-activated cell sorting (FACS) buffer containing 0,5% bovine serum albumin (Miltenyi, Bergisch Gladbach, Germany), 1× Dulbecco’s-Phosphate-Buffered-Saline (Gibco), and cooled at 4 °C. Tumor samples were preserved in MACS Tissue Storage Solution (Miltenyi, Bergisch Gladbach, Germany). TILs were isolated using the Tumor Dissociation Kit and TIL isolation kit (Miltenyi, Bergisch Gladbach, Germany) according to the manufacturer’s protocol. Viable cell counts were determined using the Automated CellCounter TC20TM. Cells were transferred to an Eppendorf tube containing FACS buffer for flow cytometry. Serum fractions from blood samples were prepared and stored at −20 °C.

### 2.4. Flow Cytometry

**Figure A1:**
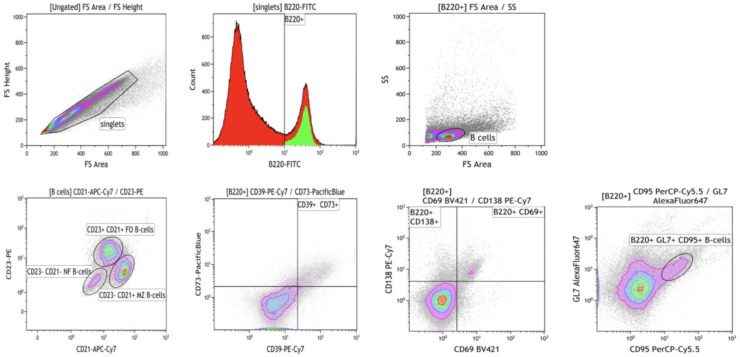
Gating strategy for the detection of B cell subpopulation in the spleen, lymph nodes and tumor tissue.

A maximum of 1 × 10^6^ cells were incubated with monoclonal antibodies (mAbs) at room temperature for 20 min in the dark. Post-incubation, cells were washed and resuspended in phosphate-buffered saline (PBS) with 0.5% bovine serum albumin (BSA) for flow cytometry analysis. Stained cells from the spleen, lymph nodes, and TILs were processed and analyzed using the GALLIOS Flow Cytometer (Beckman Coulter, Brea, CA, USA) equipped with Kaluza flow cytometry software version 1.3 (Beckman Coulter, Brea, CA, USA). The flow cytometry gating strategy is detailed in Appendix A, Figure A1. The following anti-mouse mAbs were used: CD73-Pacific Blue (Clone TY/11.8, Catalogue no. 127212, BioLegend, San Diego, CA, USA), CD69-BV421 (Clone FN50, Catalogue no. 310930, BioLegend), CD138-PE-Cy7 (Clone 281-2, Catalogue no. 142530, BioLegend), CD95-PerCP-Cy5.5 (Clone SA367H8, Catalogue no. 152610, BioLegend), CD21-APC-Cy7 (Clone Bu32, Catalogue no. 354928, BioLegend), CD39-PE-Cy7 (Clone 24DMS1, Catalogue no. 25-0391-82, eBioscience, San Diego, CA, USA), CD23-PE (Clone B3B4, Catalogue no. 12-0232-82, eBioscience); GL7-AlexaFluor 647 (Clone 1B4, Catalogue no. 560298, BD Pharmingen, Franklin Lakes, NJ, USA), B220-FITC (Clone RA3-6B2, Catalogue no. 553088, BD Pharmingen), IgM-PE (polyclonal, Catalogue no. 1021-09, SouthernBiotech, Birmingham, AL, USA).

### 2.5. ELISA

Sandwich ELISA was used to monitor Ab composition changes over a 21-day period, with sera derived from murine blood collected every 7 days for IgM, IgG1, IgG2a, IgG2b, and IgG3 antibodies using standard protocols. Briefly, the primary capture Ab Goat Anti-Mouse IgM (Capture Antibody 1020-01), Goat Anti-Mouse IgG1 (Capture Antibody 1070-01), Goat Anti-Mouse IgG2a (Capture Antibody 1080-01), Goat Anti-Mouse IgG2b (Capture Antibody 1090-01), Goat Anti-Mouse IgG3 (Capture Antibody 1100-01) (Southern Biotechnology, Birmingham, AL, USA) was diluted 1:500 in PBS (10 µL antibody in 4.99 mL PBS per plate). A total of 50 µL was pipetted into each well and incubated overnight at 4 °C to allow antibody coating. The next day, unbound Ab was removed by decanting and blotting, followed by the addition of blocking solution (10% FBS in PBS) per well. The plate was incubated overnight at 4 °C. On day three, after discarding the blocking solution, the plate was loaded with a serial dilution of either Standard-Ab-Mouse IgM-UNLB (Standard-Ab-Mouse 101-01), Mouse IgG1-UNLB (Standard-Ab-Mouse 102-02), Mouse IgG2a-UNLB (Standard-Ab-Mouse 103-01), Mouse IgG2b-UNLB (Standard-Ab-Mouse 104-01) or Mouse IgG3-UNLB (Standard-Ab-Mouse 105-01) (Southern Biotechnology) in designated wells, while the remaining wells were filled with appropriately diluted mouse serum. The standard solution (10% FCS (GIBCO), PBS (GIBCO), 0.05% Tween 20) was diluted seven times by a factor of 3 (1:3000 to 1:729,000) and filled to corresponding standard dilution wells of the plate.

Mouse serum was diluted threefold for each Ig (10% FCS (GIBCO), PBS (GIBCO), 0.05% Tween 20), e.g., IgM at 1:300, 1:600, and 1:1800 with similar calculations for other Ig types (IgG1 at 1:1500, 1:4500 und 1:13,500; IgG2a and IgG3 at 1:600, 1:1800, 1:5400; IgG2b at 1:900, 1:2700, 1:8100). Each dilution was tested in triplicate, using 9 wells per Ig per mouse.

On day four, the plate was washed five times, and the secondary Detection Ab Goat Anti-Mouse IgM (Detection Antibody 1020-04), Goat Anti-Mouse IgG1 (Detection Antibody 1070-04), Goat Anti-Mouse IgG2a (Detection Antibody 1080-04), Goat Anti-Mouse IgG2b (Detection Antibody 1090-04) or Goat Anti-Mouse IgG3 (Detection Antibody 1100-04) (Southern Biotechnology) was added and incubated at room temperature for 1 h. A light-sensitive Nitrophenylphosphate-Disodium in substrate buffer (Diethanoamin (Sigma-Aldrich, Taufkirchen, Germany), H_2_O (Ampuwa, Fresenius Kabi, Bad Homburg, Germany), MgCl_2_ (Fresenius Kabi), 19% NaN_3_ (Merck), HCl (Fresenius Kabi)) was freshly prepared and added to initiate a colorimetric reaction.

Absorbance and antigen concentration were measured at 405 nm with a TeCan photometer upon visible color change. Data from three experiments conducted under identical conditions were combined for analysis. Blood samples were collected via puncture of the vena facials and immediately transferred into 1.5 mL reaction tubes. After a 30-min coagulation period at room temperature, the tubes were centrifuged at 4 °C for 20 min. The serum fraction was carefully transferred into an Eppendorf tube for storage at –20 °C.

## 3. Results

### 3.1. Identification of Potentially Pro- and Anti-Tumorigenic B Cell Populations in a Mouse Model of HNSCC

#### 3.1.1. Increased B Cell Numbers in Spleen, Draining Cervical Lymph Nodes and Tumor Tissue During Tumor Progression in Orthotopic HNSCC

**Figure 1:**
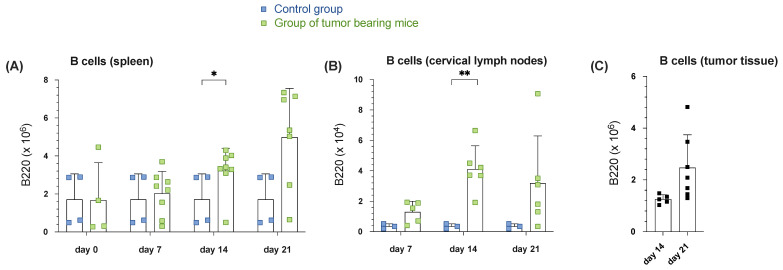
Increased B cell number in the spleen, draining cervical lymph nodes and tumor tissue during tumor progression in orthotopic HNSCC. (**A**) An absolute number of B220^+^ B cells in spleen tissue (**A**) in cervical lymph nodes (**B**) on days 0, 7, 14, and 21 was assessed by flow cytometry comparing the control group (blue point) and a group of tumor-bearing mice (green point). (**C**) The absolute number of B220^+^ B cells in tumor tissue during observation time on days 14 and 21. Each point represents data from a single animal. Data in the graphs are shown as means ± SD (n ≥ 3 mice per control group and n ≥ 4 mice per group for tumor-bearing mice; differences in group size due to discontinuation ahead of schedule). Data are merged from at least two independent experiments. *p*-values were determined using a two-tailed Student’s *t*-test or Mann-Whitney-U test. Marked *p*-values can be considered statistically significant, * *p* < 0.05, ** *p* < 0.01.

Initially, our investigation focused on examining the absolute B220^+^ B-cell count in the analyzed tissues (spleen, cervical lymph nodes, tumor tissue) over a 21-day observation period. Our findings revealed a continuous increase in B-cell presence within the spleen tissue of tumor-bearing mice over time (Figure 1A). Contrary to the abdominal lymph nodes (not depicted here for clarity), the analysis of cervical lymph nodes also demonstrated a dynamic pattern. Unlike the steady growth observed in the spleen tissue of tumor-bearing mice, the B-cell count in cervical draining lymph nodes peaked at day 14 and slightly declined by day 21, ultimately surpassing the control group significantly (Figure 1B). The B cell fraction increased compared to the constant control group (3.56 × 10^3^ ± 1.60 × 10^3^, n = 3), reaching 4.11 × 10^4^ ± 1.53 × 10^4^ on day 14 (n = 6) in the tumor group and declined to 3.19 × 10^4^ ± 3.10 × 10^4^ on day 21 (n = 6). Upon examining the tumor tissue, a continuous and nearly significant increase in absolute B cell total count was revealed in the tumor group from day 14, reaching its peak on day 21 (Figure 1C). A rise from 5.46 × 10^4^ cells on day 14 to 1.76 × 10^5^ cells on day 21 was documented in this instance. Consequently, our results underscore that a discernible increase in B-cell numbers under tumor burden was evident in the spleen, draining lymph nodes as well as tumor tissue over the course of the observation relative to the control group. These findings suggest the presence of a B cell dynamic under tumor burden, which prompted us to investigate the B cell compartment in tumor-bearing mice in more detail.

#### 3.1.2. Increased B Cell Activation, GC Formation and Plasmablast Generation During Tumor Formation in Spleen, Lymph Nodes and Tumor Tissue

**Figure 2:**
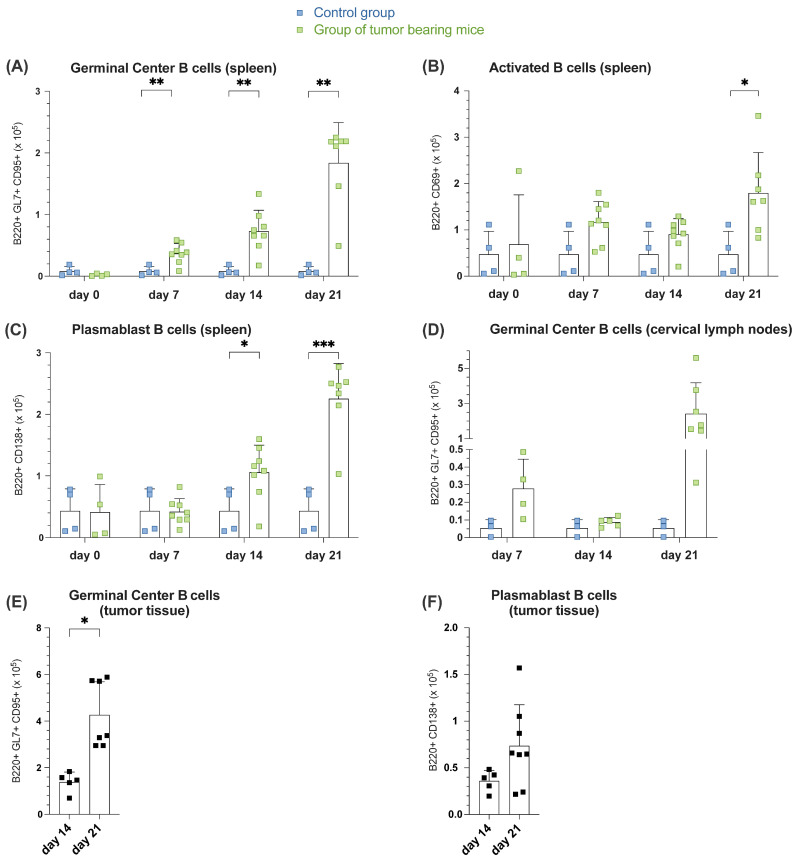
Increased B cell activation, GC formation and plasmablast generation within the time course of tumor formation in spleen, lymph nodes and tumor tissue. (**A**–**C**) Absolute number of B220^+^GL7^+^CD95^+^ GC B cells (**A**), B220^+^CD69^+^ activated B-cells (**B**) and B220^+^CD138^+^ plasmablast B cells (**C**) in spleen on days 0, 7, 14, and day 21 assessed by flow cytometry comparing control group (blue point) and group of tumor-bearing mice (green point). (**D**) An absolute number of B220^+^GL7^+^CD95^+^ GC B cells in cervical lymph nodes during observation time on days 0, 7, 14, and 21 was assessed by flow cytometry comparing the control group (blue point) and a group of tumor-bearing mice (green point). (**E**,**F**) An absolute number of B220^+^GL7^+^CD95^+^ GC B cells (**E**) and B220^+^CD138^+^ plasmablasts (**F**) in tumor tissue during observation time on days 14 and 21. Each point represents data from a single mouse. Data in the graphs are shown as means ± SD (n = 3 mice per control group; n = 4 for tumor-bearing mice groups, differences in group size due to discontinuation ahead of schedule). Data are merged from at least two independent experiments. *p*-values were determined using a two-tailed Student’s t-test or Mann-Whitney-U test. Marked *p*-values can be considered statistically significant, * *p* < 0.05, ** *p* < 0.01, *** *p* < 0.001.

The function of a GC is to provide a suitable environment for B cells to generate high-affinity antibodies. Regarding B220^+^CD95^+^GL7^+^ GC B cells, a continuous increase in the absolute proportion of this cell population among all B220^+^ splenocytes in tumor-bearing mice was consistently observed over the analyzed time points. A steady rise in GC B cells in the tumor group compared to the constant control cell count was noted, with significant differences observed on day 7 (3.69 × 10^4^ ± 1.67 × 10^4^, n = 8), day 14 (7.31 × 10^4^ ± 3.39 × 10^4^, n = 8) and day 21 (1.84 × 10^5^ ± 6.53 × 10^5^, n = 7) (Figure 2A). Since we observed an increase in total B cell numbers in SLOs as well as tumor tissue, we wondered whether this B cell enrichment is accompanied by B cell activation and differentiation. CD69 can be understood as an immunomodulator following B cell activation by tumor antigens, serving as an early B cell activation marker, a prerequisite for subsequent adaptive humoral immune responses. Examining B cells expressing CD69, an increased cell count compared to the control was recorded on each examination day. While the control count remained constant (4.75 × 10^4^ ± 4.95 × 10^4^, n = 4), the population in the tumor group reached its highest level at day 21, significantly (*p* = 0.0225), with an average of 1.80 × 10^5^ ± 8.71 × 10^4^ cells (Figure 2B). Plasma B cells, the most mature differentiation stage of B cells, crucially function by secreting antibodies upon activation by antigens, including tumor antigens. Analyzing B cells for the co-expression of the markers B220 and CD138 to detect plasmablasts in their early proliferative stage revealed no changes in the absolute number of this population in spleen tissue in both control and tumor groups on days 0 and 7, whereas a continuous and significantly increase was observed on days 14 and 21, reaching its peak on day 21 compared to the control (Figure 2C) (day 14: *p* = 0.0341; day 21: *p* = 0.0003). Additionally, a noticeable and nearly significant difference in GC B cell number (*p* = 0.0534) between the tumor group and the control population in cervical lymph nodes was observed (day 21; Figure 2D). More interestingly, the GC B cell fraction B220^+^GL7^+^CD95^+^ in the tumor tissue among all B-TILs increased significantly (*p* = 0.0025) from 1.39 × 10^5^ ± 4.24 × 10^4^ cells (n = 5) on day 14 to 4.27 × 10^5^ ± 1.42 × 10^5^ cells on day 21 (n = 7) (Figure 2E). Closely linked to the rise in GC B-TILs, further analysis revealed an increase in CD138^+^ plasma B cells in the early proliferative state. The cell counts nearly doubled from 3.61 × 10^4^ ± 1.12 × 10^4^ cells on day 14 (n = 5) to 7.37 × 10^4^ ± 4.39 × 10^4^ cells on day 21 (n = 8) (Figure 2F).

#### 3.1.3. Tumor-Induced GC Reaction Correlates with the Appearance of Switched Igs in Serum of Tumor-Bearing Mice of Orthotopic HNSCC

**Figure 3:**
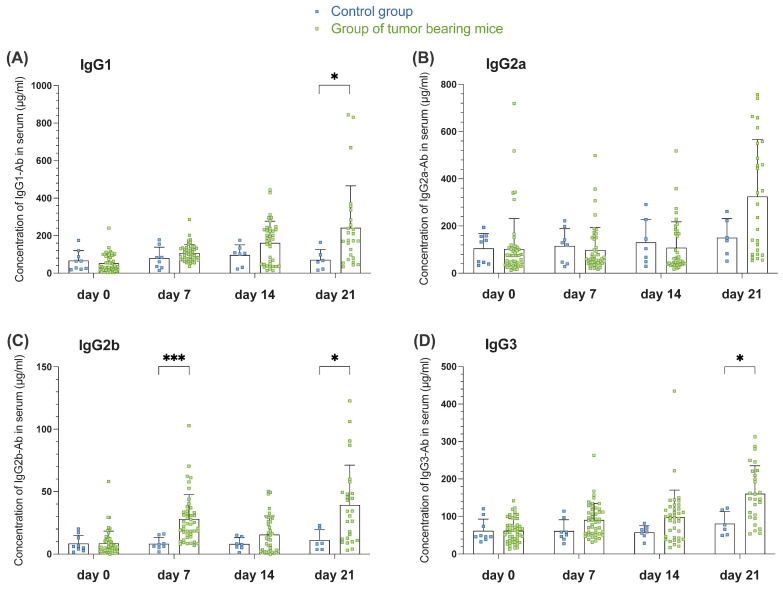
Tumor-induced germinal center (GC) reaction correlates with the appearance of switched immunoglobulins (Igs) in the serum of tumor-bearing mice of orthotopic head and neck squamous cell carcinoma (HNSCC). A statistical representation of the concentration of IgG1—antibody (Ab) (**A**), IgG2a—Ab (**B**), IgG2b—Ab (**C**) and IgG3—Ab (**D**) on days 0, 7, 14, and 21 assessed by Enzyme-Linked Immunosorbent Assay (ELISA) comparing control group (blue point) and a group of tumor-bearing mice (green point). Data in the graphs are shown as means ± SD (n = 6 mice per control group, n = 23 for tumor-bearing mice groups, differences in group size due to death due to tumor progress). Data are merged from at least two independent experiments. *p*-values were determined using a two-tailed Student’s *t*-test or Mann-Whitney-U test. Marked *p*-values can be considered statistically significant, * *p* < 0.05, *** *p* < 0.001.

Igs of the IgG type, in contrast to IgM, can only be produced by plasma cells after a class switch reaction and are divided into four IgG subclasses in mice (IgG1, IgG2a, IgG2b, and IgG3) based on their heavy chain. These subclasses circulate in the bloodstream to varying degrees and contribute differentially to complement activation as well as antigen neutralization and opsonization. Murine IgG1 antibodies are often regarded as key antibodies in Th2-mediated immune responses, capable of eliminating toxins and viruses through steric blockade. IgG1 functions in a T-cell-dependent manner through the effector function-restricting and inhibitory Fc gamma receptor IIb, thus limiting inflammatory processes and potentially excessive immune reactions [24]. Throughout the observation period, a continuous increase in the IgG1 subtype was noted. While IgG1 concentration in the control group exhibited relatively minor fluctuations with measurements of 67.21 ± 53.07 μg/mL on day 0 (n = 9), 80.10 ± 58.98 μg/mL on day 7 (n = 8), 97.09 ± 54.30 μg/mL on day 14 (n = 7), and 71.25 ± 54.77 μg/mL on day 21 (n = 6), the IgG1 serum concentration steadily increased in the tumor group over the observation period. On the final analysis day, a significant difference (*p* = 0.0139) was observed, with the tumor group (242.88 ± 222.78 μg/mL, n = 26) recording the highest average compared to the control (71.25 ± 54.77 μg/mL, n = 6) (Figure 3A). The immune response through IgG2a antibodies is enhanced in a T-cell-dependent manner. Regarding IgG2a serum concentration, a lower concentration of this Ig was measured in the tumor group than in the control group up to day 14. From this point until day 21, the Ig concentration increased to the maximum value measured in the tumor group (n = 27), reaching 324.74 ± 240.80 μg/mL compared to the control group (n = 6) with 150.98 ± 80.04 μg/mL (Figure 3B). IgG2b antibodies are part of the T-cell-independent immune response and work in conjunction with IgG3 antibodies, according to Collins et al. Additionally, activated B cells can use IgG2b as a cytotoxic element against tumor cells [24]. Unlike IgG1 (Figure 3A), the analysis of IgG2b concentration did not show a continuous but rather an undulating increasing trend in the tumor group during this observation period. After exposure of the mice to the tumor antigen on day 0, the concentration of IgG2b in the tumor group (treated group) (28.01 ± 19.42 μg/mL, n = 45) significantly (*p* < 0.0001) increased compared to the untreated and nearly constant control (8.47 ± 6.61 μg/mL, n = 9) at day 7. By day 14, the serum concentration in the tumor group (treated group) (n = 38) decreased to 15.73 ± 14.71 μg/mL, with the control concentration remaining almost unchanged (8.20 ± 4.99 μg/mL, n = 7). On day 21, a significantly (*p* = 0.0430) higher IgG2b serum concentration was observed in the treated group (39.24 ± 31.89 μg/mL, n = 27) compared to the control (11.24 ± 8.38 μg/mL, n = 6) (Figure 3C). Similar to IgG1, a continuous increase in the average IgG3 serum concentration in the tumor group (treated group) was seen throughout the entire investigation period. While minimal changes occurred in the control group, steadily increasing IgG3 serum concentrations were evident in the treated mice. By day 21, the difference between the control (80.54 ± 31.85 μg/mL, n = 6) and tumor group (treated group) (160.47 ± 74.69 μg/mL, n = 27) was significant (*p* = 0.0161) (Figure 3D).

#### 3.1.4. Increased MZ B Cell Population in Spleen, Draining Lymph Nodes and Tumor Tissue During Tumor Progression Correlates with Increased Levels of Serum IgM in Tumor-Bearing Mice of Orthotopic HNSCC

**Figure 4:**
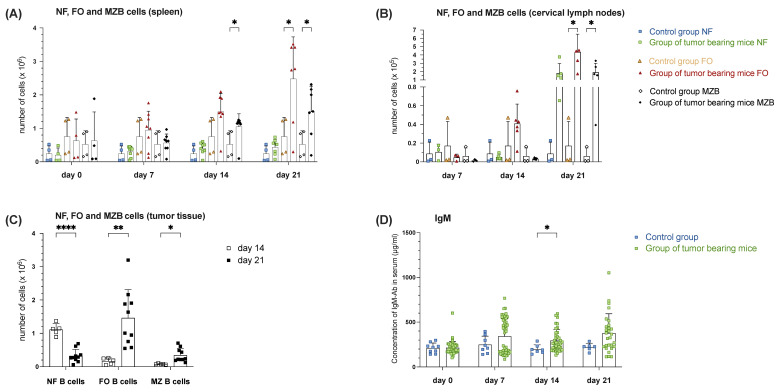
Increased MZ B cell population in spleen, draining lymph nodes and tumor tissue during tumor progression is paralleled with increased levels of serum IgM in tumor-bearing mice of orthotopic head and neck squamous cell carcinoma (HNSCC). An absolute number of newly formed B cells (NF), follicular B cells (FO) and MZ B cells in spleen tissue (**A**), in cervical lymph nodes (**B**) on days 0, 7, 14, and 21 assessed by flow cytometry comparing control group (blue point) and group of tumor-bearing mice (green point). (**C**) The absolute number of B220^+^ B cells in tumor tissue during observation time on days 14 and 21. (**D**) The concentration of IgM on days 0, 7, 14, and day 21 was assessed by ELISA, comparing the control group (blue point) and the group of tumor-bearing mice (green point). Each point represents data from a single animal. Data in the graphs are shown as means ± SD (n ≥ 3 mice per control group; n ≥ 3 for tumor-bearing mice groups, differences in group size due to discontinuation ahead of schedule). Data are merged from at least two independent experiments. *p*-values were determined using a two-tailed Student’s t-test or Mann-Whitney-U test. Marked *p*-values can be considered statistically significant, * *p* < 0.05, ** *p* < 0.01 and **** *p* < 0.0001.

Changes in the absolute proportion of NF B cells (B220^+^CD21^−^CD23^−^), FO B cells (B220^+^CD21^+^CD23^+^), and MZ B cells (B220^+^CD21^+^CD23^−^) among all measured B220+ lymphocytes in the spleen are depicted below. Characterizing the whole B220^+^ B-cell population further (Figure 1A) based on the expression level of CD21 and CD23, NF, FO, as well as MZ B cells can be distinguished. A continuous increase in NF B cells was observed in the tumor group compared to the control mice (2.44 × 10^5^ ± 2.31 × 10^5^, n = 4) over the observation period, reaching its peak on day 21 with 4.38 × 10^5^ ± 2.37 × 10^5^. Similarly, the absolute number of FO B cells in the tumor group steadily grew compared to the control (7.56 × 10^5^ ± 5.65 × 10^5^, n = 4) over the experimental period. A significant increase (*p* = 0.0307) was observed on day 21, with the largest absolute cell number of FO B cells at 2.48 × 10^6^ ± 1.25 × 10^6^ compared to the control. Interestingly, the absolute numbers of MZ B cells also increased steadily in the tumor group. Significantly more (*p* = 0.0485) of these cells were counted in the tumor group (1.05 × 10^6^ ± 3.86 × 10^5^, n = 8) than in the constant control population (5.28 × 10^5^ ± 3.98 × 10^5^, n = 4) on day 14. On day 21, the tumor group exhibited the highest absolute numbers, significantly (*p* = 0.0452) surpassing the control with 1.50 × 10^6^ ± 7.64 × 10^5^ detected (Figure 4A). Therefore, it seems likely that the increase in B cells in SLOs, as well as in the tumor over the observation period, was primarily attributable to the rise in FO and MZ B cells. Examining the absolute distribution of NF B cells among all lymph node B cells in the cervical region, the total NF B cell count in the tumor group fluctuated over the study period compared to the constant control. While more NF B cells were observed in the tumor group than the control group on day 7, the value on day 14 was below the control but increased to 20 times that of the control by day 21. The total number of FO B cells in the tumor group, however, grew continuously over the observation period and was significantly increased on day 21 compared to the control. Particularly noteworthy was the substantial increase on day 21 compared to the control group (Figure 4B). Absolute changes in the proportion of NF, FO and MZ B cells among all B cells within the tumor tissue during the observation period are presented in Figure 3B. Notably, the proportion of FO B cells increased significantly (*p* = 0.0053) from day 14 to day 21, as did the proportion of MZ B cells (*p* = 0.0129). The absolute number of NF B cells, on the other hand, decreased significantly from day 14 to day 21 in favor of the discussed increasing B cell subpopulations (Figure 4C). IgM, secreted as the first isotype by B cells, serves as the earliest parameter for an ongoing immune response, here triggered by the foreign tumor antigen. In the treated group, an increase from 214.55 ± 70.87 μg/mL on day 0 (n = 51) to an average of 343.42 ± 194.25 μg/mL was recorded on day 7 (n = 46), with 211.30 ± 56.15 μg/mL on day 0 and 249.07 ± 94.57 μg/mL in the control groups. By day 14, the IgM serum concentration in the tumor group (294.01 ± 122.29 μg/mL, n = 38) decreased, with a significant difference (*p* = 0.0109) compared to the control group (198.08 ± 49.26 μg/mL, n = 14). By day 21, the IgM in the tumor group (n = 28) reached the measured maximum average value of 376.72 ± 216.42 μg/mL (Figure 4D).

#### 3.1.5. Increased B Cell-Mediated Potential of Ado-Generation During Tumorigenesis in Orthotopic HNSCC

**Figure 5:**
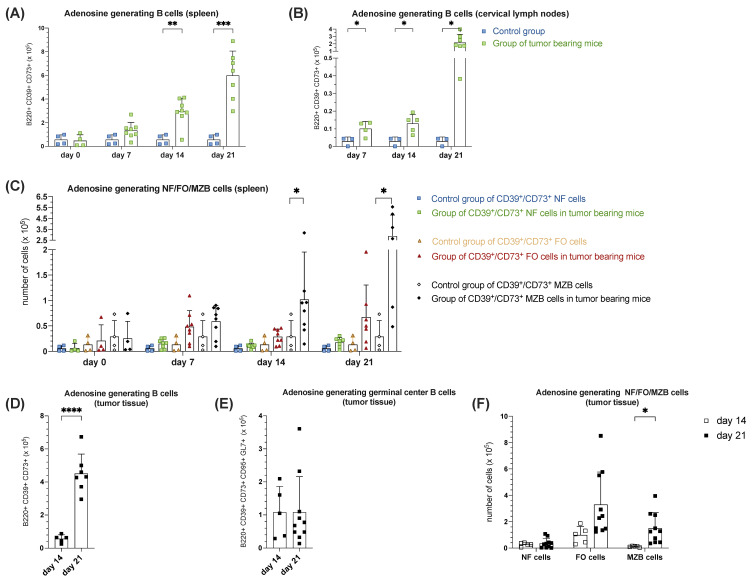
Increased B cell-mediated potential of Ado generation during tumorigenesis in orthotopic HNSCC. (**A**) An absolute number of splenic or (**B**) cervical lymph node derived B220^+^CD39^+^CD73^+^ B cells, able to generate Ado. (**C**) The absolute number of splenic CD39^+^CD73^+^ NF, FO, MZ B cells on days 0, 7, 14, and 21 were assessed by flow cytometry comparing the control group (blue, yellow, white points) and a group of tumor-bearing mice (green, red, black points). (**D**) Absolute number of B220^+^CD39^+^CD73^+^ B cells, able to generate Ado, (**E**) GC B cells, able to generate Ado (B220^+^CD39^+^CD73^+^CD95^+^GL7^+)^ and (**F**) CD39^+^CD73^+^ NF, FO, MZ B in tumor tissue during observation time on day 14 and day 21. Each point represents data from a single animal. Data in the graphs are shown as means ± SD (n ≥ 3 mice per control group; n ≥ 4 for tumor-bearing mice groups, differences in group size due to discontinuation ahead of schedule). Data are merged from at least two independent experiments. *p*-values were determined using a two-tailed Student’s t-test or Mann-Whitney-U test. Marked *p*-values can be considered statistically significant, * *p* < 0.05, ** *p* < 0.01, *** *p* < 0.001, and **** *p* < 0.0001.

Ado-generating B cells were identified according to their expression of the ectonucleotidases CD39 and CD73, converting sequentially ATP to adenosine diphosphate (ADP), adenosine monophosphate (AMP) and finally to Ado [15]. The analysis of co-expression of the surface markers CD39 and CD73 on B220^+^ cells in the spleen on days 7, 14 and 21 showed a significant, continuous increase in the expression of these ectonucleoside triphosphatase/diphosphohydrolases in the tumor group compared to the control group. Thus, a continuous and, compared to the control from day 14 onwards, significantly increasing trend in the average B220^+^CD39^+^CD73^+^ B cell count with a peak on day 21 with 5.99 × 10^5^ ± 20.63 × 10^4^ was recorded over the observation period (Figure 5A). Analyzing splenic NF, FO and MZ B cell populations solely based on CD39^+^CD73^+^ surface expression (Figure 5B), little difference between tumor and control groups in the respective cell populations could be discerned on days 0 and 7. However, particularly on day 14, a significant increase (*p* = 0.0485) in the MZ B cell population in the spleen tissue of the tumor group compared to the control was highlighted. On day 21, the greatest difference between the MZ B control and MZ B tumor group was detected (*p* = 0.0288) (Figure 5B). Similar to the spleen, a continuous increase in the significance of the absolute B220^+^CD39^+^CD73^+^ B cell counts in cervical lymph nodes over the observation period was detected in the tumor-bearing mice group compared to the control (Figure 5C). The same effect we observed within the TME. Regarding the total number of B220^+^CD39^+^CD73^+^ cells in the tumor, a fourfold significant (*p* < 0.0001) increase from day 14 (5.78 × 10^4^ ± 2.31 × 10^4^ cells, n = 5) to day 21 (4.51 × 10^5^ ± 1.19 × 10^5^ cells, n = 7) was detected (Figure 5D). When examining the NF, FO, and MZ B cell populations based on CD39^+^CD73^+^ surface expression in tumor tissue, an absolute increase in the CD39/CD73 double-positive surface expression was observed in each of these cell populations. Particularly, the proportion of MZ B cells with Ado-generating properties was thirteenfold higher and therefore significantly (*p* = 0.0218) increased on day 21 compared to day 14, whereas the CD39/CD39 double positive FO B cell population increased more than threefold within the same time frame (Figure 5E). Analyzing the CD39^+^CD73^+^ surface expression on the intratumor GC B cells, there was little difference in absolute terms during the observation interval (Figure 5F). Therefore, we concluded that CD39^+^CD73^+^ MZ B cells represent the majority of potentially Ado-generating B cells during tumorigenesis within the TME as well as in SLOs.

## 4. Discussion

### 4.1. Increased B Cell Numbers in Spleen, Draining Lymph Nodes, and Tumor Tissue During Tumor Progression

Previous research mainly focused on T cells for head and neck anti-tumorigenic function. However, recent studies documented a positive correlation between the presence of TME-associated B cells and overall survival [27]. This study aimed to analyze changes in the B cell compartment in SLOs and the TME. During tumor progression, increased B cell infiltration was observed in the spleen, cervical lymph nodes, and tumor tissue compared to controls. Our data suggest that this increase in spleen B cells was primarily due to higher proportions of FO and MZ B cells, while the percentage of NF cells decreased [28]. Like findings by Murphy et al. [28], FO B cells represented the largest subgroup of B cells in spleen tissue and reached the greatest difference between tumor and control groups on day 21. FO B cells, essential for T cell-dependent humoral immunity, likely contribute to elevated GC B cells and Ab production against tumor antigens by differentiating into long-lived plasma cells through T cell-dependent B cell responses involving Ig isotype switching, somatic hypermutation, and affinity maturation, capable of producing highly specific antibodies against tumor-associated antigens. Consistently, dynamics in Ig development, ranging from IgM to various IgG antibodies, were observed. Therefore, the increased proportion of FO B cells detected here may contribute to the elevated proportions of detected GC B cells in the spleen tissue of tumor-bearing mice.

In contrast to the spleen, where a continuous increase in B cell count was observed over time, the cervical lymph nodes exhibited a more nuanced dynamic. In cervical lymph nodes of tumor-bearing mice, B cell numbers peaked at day 14 and then declined by day 21. Firstly, a delayed immune response might occur because the immune system needs time to react to tumor antigens, involving antigen recognition, immune cell activation, and proliferation, potentially causing an initial rise in B cell count. Furthermore, the immunosuppressive TME, particularly in HNSCC, could delay the immune system’s response to the escalating tumor burden. However, upon recognition of tumor antigens, the immune system may become overactivated, resulting in a significant proliferation of B cells. Nevertheless, this excessive response may subsequently exhaust the immune system, leading to a decline in B cell count from day 14 to day 21. Additionally, direct or indirect effects of the tumor burden may influence the B cell count. High tumor burden might initially stimulate the immune system, followed by a reduction in B cell count as the tumor overwhelms the immune system, possibly explaining the decrease from day 14 to day 21. This decline in B cells in cervical lymph nodes could indicate cellular migration towards the tumor to function as B TILs with anti-tumoral activity. Similar to Ruffin et al.’s analyses on HNSCC [29], we observed over 60% B TILs during the observation period, supporting the hypothesis of migrating B cells from cervical lymph nodes infiltrating the TME. Consequently, an increased presence of B TILs might enhance intra-tumoral antigen presentation to CD4^+^ T cells, potentially resulting in an intensified intra-tumoral tumor antigen-adapted Ab response later on.

Similar to the spleen, a continuous increase in B cell infiltration within the tumor tissue was detected in the TME during HNSCC progression. Tumor growth alters the TME, including cytokine and chemokine profiles and the cellular and extracellular environment [8,11,30], fostering the recruitment and homing of both pro- and anti-tumoral B cells within tumor tissue [31]. Some B cell subtypes may exhibit anti-tumoral functions by producing cytotoxic antibodies, activating T-cell responses, or forming TLS, while others may have immunosuppressive properties [31]. The precise composition of the TME and the functionality of TME cell components, particularly regarding their pro- or anti-tumorigenic potential, remain insufficiently explored in HNSCC. Therefore, we examined B cells in the aforementioned tissues in more detail to further identify corresponding pro- and anti-tumoral subgroups based on their phenotype.

### 4.2. GCs and Their Function

The rising number of FO B cells in the tumor group compared to the control in spleen tissue was accompanied by an increasing number of activated CD69^+^ B cells, GC B cells, and plasma cells. The rise in CD69^+^ B cells in both spleen and tumor tissue indicates an ongoing B cell activation and clonal expansion in the context of generating an adaptive immune response. Zimmermann et al. showed that prolonged antigen exposure to B cells increases CD69 expression [32], while Maddur et al. found that antigen presentation by follicular dendritic cells enhances CD69 expression on B cells, regulating DZ migration in lymph nodes [33]. This activation is crucial for enhanced antigen presentation to CD4^+^ and CD8^+^ T cells intratumorally, as well as in lymph node and spleen tissues and for differentiation into active tumor antigen-specific Ab-secreting B cells [34]. The increase in CD69^+^ B cells corresponded with a tremendous rise in the absolute proportion of B220^+^CD95^+^GL7^+^ GC B cells among all B220^+^ cells in tumor-bearing mice in lymph nodes and spleen throughout the observation period. The significant increase in GC B cells in tumor tissue from day 14 to day 21 suggests a persistent B cell-mediated anti-tumor immune response in the spleen, draining lymph nodes and tumor tissue itself.

Furthermore, the presence of GC B cells in tumor tissue implies the formation of TLS [29,35], which is associated with GC formation and gene expression involved in adaptive immune response. TLSs are transient, ectopic lymphoid structures found in chronically inflamed tissues, acting as modulators of anti-tumoral immune responses [36]. These mature ectopic vascularized lymphoid structures resemble SLOs. The aggregation of various TILs within a developed TLS facilitates the effective presentation of tumor-associated antigens and subsequent T and B cell effector activity [37]. Positioning themselves in the immediate tumor environment or within tumor tissue itself, TLSs enable direct antigen stimulation in proximity to the tumor, local lymphocyte recruitment, and circulation at the invasive tumor margin, enhancing tumor infiltration by anti-tumoral immune cells [38,39]. GC growth parallels TLS development, eventually maturing into activated GCs. Researchers have linked different TLS subtypes to patient survival [40]. Thus, GC formation in TLSs is critical for tumor development and treatment.

Although researchers have identified various TLS subtypes, no universal classification exists. In colorectal cancer (CRC), Posch et al. proposed three TLS subtypes based on FDC and mature B cell density, resulting in GC reactions [41]. Hiraoka et al. conducted a location-based evaluation of TLS and reported that intratumoral TLS correlated with better patient outcomes [40], linked to an active immune response and a well-developed vascular network in the TME. Unlike Werner et al.’s findings in malignant melanoma [42], we did not find classical TLS intratumorally. However, our studies showed increasing GC B cells and ongoing GC reactions, along with long-lived plasma cells arising from the GC reaction, suggesting the development of secondary follicular-like TLS in HNSCC.

Our analysis revealed increased levels of GC B cells and plasmablasts, supporting our notion of GC-like structures in the used mouse model of HNSCC, which were described previously in human HNSCC as predictive for the patient’s outcome [29,43,44,45,46,47]. Effector B cells differentiate into plasma and memory B cells, producing long-lasting immune responses with specific anti-tumoral antibodies [36]. We demonstrated that mature plasma cells (CD138^+^B220^+^) increased towards the end of the experiment in both spleen and tumor tissues, peaking on day 21 with the highest immunohistochemically demonstrated GC formation and Ab titers produced by plasma cells. Plasma cells release antibodies against TAAs, making them crucial anti-tumor effector B cells, either aggregating within TLS interstitium or dispersing in the tumor stroma, forming cell clusters [34,47,48,49]. Kröger et al. showed that plasma cells enhance CTL cytotoxicity [49]. Concomitant with increasing tumor burden in the tumor group, we also noted an increase in CD8^+^ T cells in the spleen, cervical lymph nodes, and intratumorally, potentially relating to higher plasma B cell counts and anti-tumoral activity.

Wouters et al. showed that B-TIL cells or plasma cells coexisting with T cells significantly improve prognostic relevance in various cancer entities [50]. Although we did not detail affinity maturation, our FACS analysis and increase in antigen-specific IgG antibodies suggest that prolonged exposure to tumor antigens leads to more affinity-matured and class-switched B cells. These B cells likely recognize tumor antigens and develop into active CD138^+^ plasma cells or memory cells, establishing ongoing humoral immunity. The significant rise in GC B cells with tumor growth indicates the immune system’s effort to create anti-tumoral immunity near the inflammation site so that it can act promptly. This proximity allows for efficient antigen presentation, B cell activation, GC reactions, and localized Ab production. However, due to the immunosuppressive nature of HNSCC, this immune response may not fully unfold.

### 4.3. Tumor-Induced GC Reaction Correlates with the Appearance Ig Switched Immunoglobulins in Serum of Tumor Formation

The development of GCs in solid tumors signifies an active immune response, ultimately leading to class-switched Ig production and secretion [36,51,52]. The dynamics of Ig secretion in HNSCCs are largely unexplored, making these Ig analyses initial data on Ab production upon tumor initiation.

Murine IgG is classified into four subclasses: IgG1, IgG2a, IgG2b, and IgG3, with IgG2c being equivalent to IgG2a in some strains like C57BL/6 mice. Each subclass mediates effector functions differently due to varying specificity and affinity for IgG binding partners [53,54]. Previous mouse experiments have shown these subclasses are produced during bacterial and viral infections, DNA vaccinations, or exposure to Plasmodium antigens [55]. We demonstrated that tumor antigens induce an IgG immune response in mice. In tumor-bearing mice, we observed increased activated and GC B cell numbers in the spleen, draining lymph nodes, and the TME. This correlated with elevated IgG subtype levels over time. Initially, there was a sharp increase in IgM concentration in murine serum, possibly due to the inflammatory response from tumor implantation or Ab production by MZ B cells to protect against tumor antigens [56]. While IgM levels remained elevated but constant from day 7 onwards in the tumor group, a subtype-dependent change in IgG concentration was observed. Collin et al. proposed that the coordinated expression of all four IgG subclasses is crucial for murine IgG function [55]. Due to limited somatic hypermutation, murine IgG responses have a relatively low affinity, requiring more Ab-producing cells to eliminate antigens [55]. This necessitates larger B cell populations and an increase in plasma cells for sufficient Ab secretion, observed during tumor growth in the spleen and tumor tissue of the tumor group compared to controls. According to Collins’ model, IgG2a antibodies strengthen the immune response with T cell assistance [55]. Our analysis shows IgG2a formation in the tumor group occurring late, peaking at day 21, contradicting Collin et al.’s suggestion that IgG2a is limited to antiviral responses [55]. IgG2b, typically part of the T-independent response, was elevated early in the experiment, as they can act similarly to IgG3 in terms of T cell independence and then declined by day 14, similar to Collin et al.’s findings [55]. In contrast, IgG3 continuously increased throughout the experimental period in the tumor group, peaking on the last analysis day. According to the Collins model, murine IgG3 antibodies ensure early activation of IgG-mediated effector functions in a T-cell-independent immune response [55]. Bivalent murine IgG3 antibodies fix complement and trigger inflammatory events. Elevated serum IgG3 levels in tumor-bearing mice, even in early stages without sufficient CD4 T cell help, indicate their T-cell-independent action. Murine IgG1 antibodies, although relatively unmutated compared to human antibodies, have the highest antigen-binding affinity among murine IgG subclasses and are key in Th2-mediated responses, eliminating toxins and viruses through steric blockade. However, IgG1 cannot activate the complement cascade like IgG3 or IgG2b [55]. IgG1 acts T cell-dependently via FcγRIIb, limiting inflammatory processes and excessive immune responses [55].

Only when an efficient GC reaction is taken together can secondary Ig be produced. Betzler et al. demonstrated in a mouse model with BOB.1/OBF.1 deletion in GC B cells that impaired GC formation resulted in significantly reduced levels of IgM and switched Ig after both primary and secondary immunizations [57,58]. Thus, Igs serve as indicators of functional GC reactions in HNSCC. The presence of intratumoral GC B cells, which enable the GC reaction, suggests the generation of intratumoral anti-tumor immunity in HNSCC.

### 4.4. Increased MZ B Cell Population in Spleen, Draining Lymph Nodes and Tumor Tissue During Tumor Progression Correlates with Increased Levels of Serum IgM in Tumor-Bearing Mice

Solid tumors utilize high concentrations of extracellular ATP to create an immunosuppressive environment through enzymatic degradation of ATP by CD39 and CD73 to Ado, supporting tumors to evade the host’s immune response. This immunosuppressive milieu prevents immune cell activation and downregulates the immune system, contributing to tumor growth.

Surprisingly, we observed an increasing surface expression of CD39^+^CD73^+^ on MZ B cells under tumor progression, particularly evident in the splenic tissue of tumor-bearing mice and the tumor tissue itself compared to controls. Consequently, we postulated that MZ B cells not only act in an anti-tumoral capacity by secreting IgM short- to mid-term but also regulate possibly significantly pro-tumoral processes due to their strong immunosuppressive potential, as evidenced by CD39^+^CD73^+^ co-expression, particularly pronounced in tumor-bearing mice’s splenic tissue, cervical lymph nodes, and tumor tissue. Thus, here we described for the first time the presence of MZ B cells in the TME of HNSCC, noting their pro- and anti-tumoral properties. Also, the expression of CD39 and CD73 by MZ B cells, as well as their precursors, was described earlier by Doyon-Laliberté et al., supporting their role in Ado-mediated immunosuppression [59]. On the other hand, Ado is crucial for B cell development and immune responses, including GC reaction and plasma cell generation [60]. Together, these findings indicate an important role of MZ B cells in the regulation of immune homeostasis but also their pro-tumorigenic potential. Therefore, further research is necessary to comprehend the consequences of CD39/CD73 expression on B cells in the TME of HNSCC, a recently revealed phenomenon [12,15].

Together, our results revealed B cells within the TME of HNSCC with potentially pro- and anti-tumoral properties. Given that the presence of B cells in the TME correlates with the survival and outcome of cancer patients [24], further studies should explore specific B cell subpopulations for their capability to serve as targets for immunotherapeutic interventions in solid cancers, including HNSCC.

### 4.5. Limitations of the Study

This study provides valuable insights into immune dynamics under tumor burden, yet it is not without limitations. These constraints, detailed below, highlight areas that require further investigation to deepen our understanding and validate the findings. To investigate B cell responses during the development of HNSCC, we have applied the syngeneic murine immunocompetent SCC VII model [16] widely used to study HPV-negative oral cavity squamous cell carcinoma. Although this model offers a broad range of opportunities to study cancer-immune cell interactions, immune cell infiltration into the tumor and immune-cell-mediated anti-cancer responses, a direct translation into the human system should be treated with caution and requires further studies in appropriate patient cohorts under relevant clinical settings. However, like in patient tumors, our study revealed antibody-secreting cells [26] and also B cells, which are potentially able to generate Ado due to their expression of CD39 and CD73 and thus able to suppress the anti-tumor immune response [15]. What limits our study is the short observation period of 3 weeks after tumor growth initiation. So, we have no clue about the dynamics of the immune cell composition over a longer time.

A notable limitation is the small sample size in the control group, which may reduce the statistical power and generalizability of the findings. To comply with the 3R rules (replace, reduce, refine), the control group of 3 and 4 animals was kept small, as they are genetically identical animals. In the experimental groups, there were premature discontinuations according to our discontinuation criteria. The number of groups studied also constrains the ability to draw robust conclusions across a broader spectrum of immune and tumor characteristics. Future studies with larger cohorts and diverse groups are essential to confirm these observations.

The present study does not cover the analyses of all cell populations within the tumor microenvironment but rather focuses on the analyses of B cell-mediated immune responses. However, the tumor microenvironment is highly complex, involving interactions among a diverse array of cell types, including T cells, neutrophils, stromal cells, and endothelial cells. A more comprehensive analysis encompassing all cellular components is needed to fully understand the dynamics and functional roles of the tumor microenvironment.

This study focused on antibody detection in peripheral blood, reflecting systemic immune activation. While this approach provides insights into global immunological changes, it does not capture the dynamics of local immune responses within the tumor microenvironment itself or adjacent tissues. Future investigations should integrate analyses of local antibody production and immune activities to provide a more holistic understanding. While the study contextualizes findings with timing and immune changes, it does not include peripheral blood analysis. This absence limits the ability to directly relate murine findings to non-invasive diagnostic or monitoring strategies in clinical settings. Future studies should aim to develop assays that link peripheral blood biomarkers with immune and tumor dynamics observed in the current study. These limitations emphasize the need for broader and more integrative research approaches. By addressing these gaps, future studies can expand upon the current findings to provide a more comprehensive understanding of immune dynamics in cancer.

## 5. Conclusions

Analyzing B-TILs in a mouse model of HNSCC, we observed an increase in GL7^+^CD95^+^ GC B cell numbers and CD39^+^CD73^+^ B cells in tumors and spleens. Parallel to increased MZ B cell numbers, IgM Ab levels in mouse serum rose continuously, while IgG1, IgG2, and IgG3 levels increased later, as expected. CD39^+^CD73^+^ expression was predominantly observed on MZ B cells, suggesting an immunosuppressive potential. Tumor antigen exposure induced GCs, promoting anti-tumoral immune structures in spleen and tumor tissues. Understanding the complex interactions between B cell subsets and the TME may lead to new strategies for enhancing the treatment and prognosis of HNSCC patients.

## Data Availability

The data presented in this study are available on request from the corresponding author.
